# Delineating the Immuno-Dominant Antigenic Vaccine Peptides Against gacS*-*Sensor Kinase in *Acinetobacter baumannii*: An *in silico* Investigational Approach

**DOI:** 10.3389/fmicb.2020.02078

**Published:** 2020-09-08

**Authors:** A. S. Smiline Girija

**Affiliations:** Department of Microbiology, Saveetha Dental College and Hospitals, Saveetha Institute of Medical and Technical Sciences (SIMATS), Saveetha University, Chennai, India

**Keywords:** *A. baumannii*, gacS, sensor kinase, epitope peptides, *in silico*

## Abstract

**Objectives:**

To predict the novel vaccine peptide candidates against gacS protein involved with the citrate utilization in the two-component system of *A. baumannii*-associated virulence as an alternative strategy to combat the multi-drug resistant strains using an immuno-informatic approach.

**Methods:**

The study is designed as an observational *in silico* study design with the application of BepiPred, AlgPred, VaxiJen, AntigenPro, SolPro, Expasy ProtParam server, IEDB database, and MHC cluster analytical tools and servers to predict the immuno-dominant B-cell and T-cell epitopes from gacS FASTA sequences retrieved from UNIPROT database. Further peptide interactions with TLR-4 was assessed based on the number of hydrogen bonds.

**Results:**

Nine peptides (20aa) with the highest score of 1 were selected from the 137 epitopes, and five were predicted as antigenic epitopes (E1–E5). E3 was selected as the potent antigen (score: 0.939537) and E1 as the best vaccine candidate (score: 0.9803) under AntigenPro and Vaxijen server, respectively. SolPro predicted all epitopes as soluble peptides. ProtParam predictions showed E3 and E5 as stable proteins with a shelf life of 3.5 and 1.9 h and possessed negative GRAVY values. PsortB server predicted a final localization score of 7.88 for the gacS protein sequence as a cytoplasmic membrane protein. IEDB conservancy analysis showed 100% conserved sequences within the gacS sequence, and class I conservancy yielded positive values for all epitopes. Cluster analysis showed strong interactions, and the protein-peptide interactions with TLR-2 finally detected E5 as the best interacting peptide (H bonds = 14) followed by E3 (H bonds = 12).

**Conclusion:**

The study suggests five antigenic peptides as promiscuous vaccine candidates to target the gacS of *A. baumannii* using immuno-informatic approach toward the peptide synthesis and *in vitro* analysis. However, the study recommends further experimental validation for immunological response and memory through *in vivo* studies.

## Introduction

*Acinetobacter baumannii* is a gram-negative non-motile coccobacillus, phenotypically negative for oxidase and positive for catalase (Murray et al., 2016), that belongs to the family of *Moraxellaceae* (Baumann et al., 1968). The genus *Acinetobacter* is highly diverse and encompasses nearly 50 species, the majority being saprophytic and non-pathogenic (Al Atrouni et al., 2016) and the most potent pathogenic species comprising *A. baumannii*, followed by *A. calcoaceticus* and *A. lwoffii* (Dijkshoorn and van der Toorn, 1992). Earlier infections with *Acinetobacter* occurred between the 1960s and 1970s, as a low priority pathogen with low virulence (Rice, 2008), but in the twentieth century, multivariate analysis of the clinical isolates has documented *A. baumannii* as the most virulent pathogen (Maragakis and Perl, 2008). In addition, the World Health Organization (WHO) has declared *A. baumannii* as a priority pathogen (Chusri et al., 2014) and is also been incorporated under the ESKAPE group of pathogens (World Health Organization [WHO], 2014). The main reason behind these declarations relies on its ability to cause recalcitrant nosocomial infections such as ventilator associated pneumonia; skin and traumatic infections; urogenital and catheter-associated infections; and complicated septicemia (Rice, 2008). Multi-drug resistance (MDR), extensive drug resistance (XDR), and total drug resistance (TDR) synergistically severe the systemic infections in association with other risk factors and comorbidities (Garnacho et al., 2003). Reports from India, have documented MDR strains of *A. baumannii* (Smiline Girija et al., 2017, 2018a,b, 2019a,b) along with resistance profiles mediated by plasmids. This major sort of transformation from a low-priority pathogen into a predominant nosocomial pathogen can also be related to various virulence factors in addition to antibiotic resistance (Liu et al., 2018).

*A. baumannii* challenges itself to survive in harsh hospital environmental niches, enduring in a varying atmosphere of antiseptics, desiccating agents, temperatures, and, finally, the host biotic habitat from an abiotic habitat (Rajamohan et al., 2010). It is thus exciting to note that *A. baumannii* adapts to unfavorable conditions in a very sensible manner using the two-component systems (TCS) that regulate their phenotypes with different underlying mechanisms. With a high degree of genomic conservations, an overview of TCS shows nearly 20 operon/genes encoding for vital proteins efficiently associated with the virulence and resistance of *A. baumannii* (De Silva and Kumar, 2019). GacSAmong many TCS in *A. baumannii*, gacS is mainly related to the utilization of citrate as the sole carbon source in the citrate metabolism. It is also fascinating to observe that gacS could function as a hybrid sensor kinase based on many sequential studies, as there is no probable linking to a regulator encoding gene, and its organization may vary among different phenotypes (Dorsey et al., 2002). A clear understanding of the gacS function was best achieved through gacS mutant-related studies. The gacS mutant phenotype of *A. baumannii* could not significantly inhibit yeast (Peleg et al., 2008), attenuated the virulence in the mouse model, and insignificantly regulated the pili synthesis, motility patterns, formation of biofilms, and resistance patterns (Cerqueira et al., 2014). Additionally, gacS directly influenced the neutrophil chemotaxis to the site of infection with its vital role in the phenyl acetic acid pathway (Bhuiyan et al., 2016).

GacS-associated TCS, therefore, portray a vital role in *A. baumannii* adaptive responses and its modulations toward the antibiotic susceptibility and virulence mechanisms. Targeting gacS would be a novel approach to combatting *A. baumannii* infections that impedes last-resort treatment strategies. Vaccine strategies employing bacterial outer membrane complex and biofilm-associated proteins have entered clinical trials, however, the results are not promising and have resulted in no licensed vaccines against *A. baumannii* yet, despite numerous reports on the subject (Chiang et al., 2015). In this context, aiming for efficient prophylaxis measures, genomics- and proteomics-based new vaccine strategies, such as subtractive proteomics and reverse vaccinology methods, have been successful in identifying probable vaccine candidates (Christensen et al., 2013). The initial step in the design of synthetic peptides being the identification of potential epitopes, the present investigation is thus aimed to unravel the antigenic and immunogenic peptides from gacS of *A. baumannii*, using an immuno-informatics approach.

## Materials and Methods

### Selection of *G*gacS for Epitope Prediction

FASTA sequence of gacS from the clinical strain of *A. baumannii* (NCBI taxonomy ID 470) was retrieved from the UniProt database^1^. The UniProtKB identifier of gacS was Q8RMF4 (Q8RMF4_ACIBA) submitted under the name *Bar A* with the molecular function of phosphoryl sensor kinase activity and the subcellular location as an integral component of transmembrane protein.

#### B-Cell Epitope Mapping

Retrieved FASTA sequences of gacS were used as the input in BCPred server^2^, AAP Prediction tool that uses paired amino acid antigenicity scale (Chen et al., 2007), with the BepiPred-2.0 server, to sequentially predict the epitopes and non-epitope amino acids from the crystal structures using random forest algorithm software (Larsen et al., 2006) and an ABCPred server to predict the B-cell epitopes based on a recurrent neural network using fixed length patterns (Saha and Raghava, 2006b). The position of the nine epitope peptides with maximum threshold value of 1.0 as predicted with the AAP prediction tool (default set as > 0.5) was further selected for immunogenic analysis.

#### Predictions on Antigenicity

The predicted protein sequences from gacS were uploaded in VaxiJen v2.0 server (Doytchinova and Flower, 2007b), which can detect the protective antigens based on alignment-independent predictions to categorize the antigens according to their physicochemical properties. The output is obtained as a statement of protective antigens or other antigens according to a predefined cutoff together with the prediction probability with the precision varying from 70–80% (Doytchinova and Flower, 2007a). Additionally, prediction of the antigenic peptide was achieved by ANTIGENpro server using the reactivity data (Magnan et al., 2010).

### Analysis of the Physicochemical Properties of the Predicted Proteins

For the predicted peptides from gacS, the computation of various physical and chemical parameters was done by the ProtParam server to evaluate the molecular weight, theoretical pI, amino acid composition, atomic composition, extinction coefficient, estimated half-life, instability index, aliphatic index, and grand average of hydropathicity (GRAVY) (Gasteiger et al., 2005).

#### Predictions of Allergenic Properties

A systematic approach to predicting allergenic proteins from gacS with high accuracy was achieved using AlgPred software (Saha and Raghava, 2006a). Different approaches – such as the similarity of known epitopes with any region of protein, IgE epitope mapping, MEME-MAST allergen motif predictions, SVM modules based predictions, BLAST allergen representative peptide (ARPs) search (2890 allergen), and finally a hybrid option of combined approach (SVMc + IgE epitope + BLAST ARPs + MAST) – were employed in combination to predict the allergenic peptides.

### Signal Location of the Epitope Peptides

For verification of the gacS peptide signals and their location on *A. baumannii*, SignalP 4.1 software was used to assess the sequence options with or without transmembrane sets based on two neural networks (Petersen et al., 2011). Further, for the subcellular location prediction, PSORTb 3.0.2 was used, which functions in multiple analytical modules, each peptide being analyzed and evaluated as to how it aids the drug delivery systems (Yu et al., 2010).

### Prediction of Continuous Antibody Epitopes

As an empirical rule the position of the continuous epitopes from gacS based on parameters such as hydrophilicity, flexibility, accessibility, turns, exposed surface polarity, and antigenic propensity of the predicted antigenic peptides were achieved with Immune Epitope Database and Analysis (IEDB server). Predictions were done on propensity scales for each of 20 amino acids and the score for a given residue *i*, a window size *n* was computed with *i –* (*n −* 1)/2 neighboring residue analysis on the propensity scales. The graphs were obtained with six prediction tools, i.e., BepiPred linear epitope predictions, BepiPred 2.0 sequential epitope predictions, Karplus-Schulz flexibility predictions, Chou-Fasman beta turn predictions, Kolaskar and Tongaonkar antigenicity predictions, Emini surface accessibility predictions, and Parker hydrophilicity predictions (Chou and Fasman, 1978; Emini et al., 1985; Karplus and Schulz, 1985; Parker et al., 1986; Kolaskar and Tongaonkar, 1990).

### Predictions of T-Cell MHC Class-I and MHC Class-II Epitopes

The predicted antigenic epitopes from gacS were further subjected for MHC class I binding using the IEDB-AR server. IEDB predictions use a default and consensus calculations for predictions based on ANN, SMM, and CombLib in addition to NetMHCpan-EL, and the choice of selection was based on the decreasing order, i.e., Consensus > ANN > SMM > NetMHCpan EL > CombLib. The predicted antigenic epitope sequences were analyzed against the frequently occurring alleles set by default to occur in at least 1% of the human population. Final selections were made according to percentile ranks and the three different categorizations of the binding affinity upon the IC_50_ values.

### Predictions on Class-I Immunogenicity and Conservancy Analysis

Assessing the ability of the selected gacS peptides to evoke an immune response is considered the crucial step in immunogenicity prediction, and using the default parameters of the IEDB server, the epitopes that rendered the positive values were considered potent immunogens. Further, the conservancy of the epitopes predicted within the protein sequences is yet another measure of validating the epitopes. This was achieved by using an IEDB conservancy analysis server, and by setting the parameters at the default, the degree of conservancy was calculated.

### Cluster Analysis of the MHC Restricted Alleles

The functional relationship of the predicted peptides of gacS with the HLA alleles was inferred using the MHC cluster 2.0 server, which clusters the MHC alleles with the appropriate chosen peptides. The relationship can be assessed using the output graphical tree and static heat map obtained between the clusters.

### Interaction Between Proteins and TLR2 Receptor

To explore possible interactions of the predicted epitopes with the TLR4 receptor, a Galaxy web server was employed to assess protein-peptide binding. This step is crucial in verifying those interactions in order to progress to the design of an efficient vaccine, where the structure and optimization of the design are possible, based on the energy obtained according to the interpretation of the interactions. The number of hydrogen bonds formed between the complexes was recorded and interpreted.

## Results

### Determination of B-Cell Epitopes

The FASTA sequence of gacS of *A. baumannii* (UniProt ID: Q8RMF4) upon peptide mapping by BCPred, AACPred, BepiPred, and ABC Pred yielded 137 epitopes (Default threshold > 0.5) (Supplementary Tables 1–3). The nine peptides that had the highest score of 1 by AAC pred were selected for further analysis of antigenicity and immunogenicity (Table 1).

**TABLE 1:** AAP predictions of the possible epitopes showing maximum threshold values based on amino-acid paired antigenicity scale (Threshold value > 0.5).

Position	Epitope sequence	Score
868	LYGATRYVGTPKLQQVTGDF	1
254	HTEQTEEDLRRTLDTLEVQN	1
149	TAGKPPVWLLIEMDNQPLEL	1
495	HGQIGFEDNQERAPTEKGST	1
455	SGTDRKKLFESFSQGDASVT	1
721	QMPVMSGIDTTRAIRSLEST	1
582	KDNTWLIVDHSGDTEALLKE	1
68	KDLYTLVELQPDEYDHAQHI	1
616	QMTLEPNMLTEYRARPLYQP	1
115	YRDNRYWPNFTQNNNFFGPI	0.996
545	HPATASVLRYYLENYQVPHI	0.811
1	MSNFNKTLSKRLRLNHAYGQ	0.302
764	LLKVGMNDYVTKPIQMEQII	0.269
384	KHIAMAFYYADNIPQQVIGD	0.203

### Vaccine Properties

#### Antigenic Potentials

AntigenPro software analyzed the nine peptides selected and showed five peptides to be potential antigens. The peptide sequence E3-HGQIGFEDNQERAPTEKGST showed the highest score, 0.939537 (Table 2). The VaxiJen server 4.0 at a threshold > 0.4 showed all the five peptides (E1–E5) as potential vaccine candidates with E1-HTEQTEEDLRRTLDTLEVQN showing the highest score, 0.9803. SolPro analysis of the solubility property of the peptides showed all the epitopes as soluble peptides with E5 and E1 observed with the highest scores at a threshold set at ≥ 0.5.

**TABLE 2:** VaxiJen-, ANTIGENPro-, and SOLPro-based determination of antigenicity and solubility of the predicted epitopes.

Peptide	Epitope designations	Peptide sequence	VaxiJen		Antigen PRO	SolPro	
			Threshold value (≥0.4)			Threshold value (≥0.5)	
1	-	LYGATRYVGTPKLQQVTGDF	0.0242	Non-antigen	0.388393	0.985151	Soluble
2	E1	HTEQTEEDLRRTLDTLEVQN	0.9803	**Probable antigen**	0.351808	0.972181	Soluble
3	E2	TAGKPPVWLLIEMDNQPLEL	0.6448	**Probable antigen**	0.403709	0.810140	Soluble
4	E3	HGQIGFEDNQERAPTEKGST	0.8975	**Probable antigen**	0.939537	0.887595	Soluble
5	E4	SGTDRKKLFESFSQGDASVT	0.6830	**Probable antigen**	0.637660	0.823625	Soluble
6	-	QMPVMSGIDTTRAIRSLEST	0.2225	Non-antigen	0.579908	0.863721	Soluble
7	-	KDNTWLIVDHSGDTEALLKE	0.1258	Non-antigen	0.543209	0.920990	Soluble
8	-	KDLYTLVELQPDEYDHAQHI	0.2341	Non-antigen	0.451481	0.861114	Soluble
9	E5	QMTLEPNMLTEYRARPLYQP	0.8632	**Probable antigen**	0.382723	0.987619	Soluble

*For significance, the phrase “Probable antigen” is indicated in bold.*

#### Allergenic Properties

**FIGURE 1:**
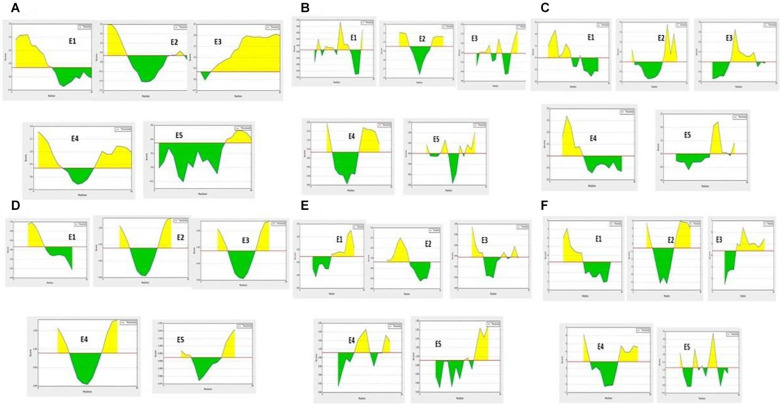
B-cell antigenic epitope predictions with the start- and end-positions showing the antigenic peptide sequences (as a yellow color peak) by **(A)** BepiPred linear epitope predictions. **(B)** Chou-Fasman beta turns assessment. **(C)** Emini surface accessibility predictions. **(D)** Karplus and Schulz flexibility predictions. **(E)** Kolaskar and Tongaonkar antigenicity. **(F)** Parker hydrophilicity assessments.

The hybrid option of the combined approach employed showed non-allergenic peptides for the IgE binding sites. MAST algorithms also showed non-allergenic peptides. E2 and E5 were finally considered as non-allergenic peptides by the hybrid approach (Table 3). Based on the antigenic and allergenic potentials, antigenic peptides E1–E5 were predicted based on biochemical properties (Figure 1).

**TABLE 3:** AlgPred predictions of allergenicity of epitopes based on SVM and hybrid approaches.

Peptide	Predicted antigens	IgE	MAST	SVM-Aa	SVM-dp	BLAST–ARP	Hybrid
E1	HTEQTEEDLRRTLDTLEVQN	NA	NA	A	A	NA	A/NA
E2	TAGKPPVWLLIEMDNQPLEL	NA	NA	NA	NA	NA	NA
E3	HGQIGFEDNQERAPTEKGST	NA	NA	PA	A	NA	A/NA
E4	SGTDRKKLFESFSQGDASVT	NA	NA	A	A	NA	A/NA
E5	QMTLEPNMLTEYRARPLYQP	NA	NA	NA	NA	NA	NA

*NA, Non-allergen; A, Allergen; PA, Potential allergen.*

### Physicochemical Analysis of the Peptides

ProtParam predictions of the five epitope peptides (E1–E5) showed two stable proteins with a shelf life of 3.5 and 1.9 h (*in vitro*). All the peptides had a MW of around 2KD with negative GRAVY values and were interpreted as hydrophilic proteins presenting strong interactions with water molecules (Table 4). With a high aliphatic index value of 117, E2 was found to be unstable but was observed to have a greater shelf life of 7.2 h. ProtParam also analyzed the isoelectric points based on the total liquid charge of the amino acid/protein as 0, on par with constant equilibrium points. It varied from 4 to 6 for E1–E5 and the stable epitopes had IP values of 4.83 (E3) and 5.84 (E5), as interpreted with a pH of 6.48.

**TABLE 4:** Physico-chemical properties of the predicted epitopes, i.e., molecular weight (MW), Isoelectric point (IP), Stability Index (SI), Shelf Life (SL), Aliphatic Index (AI), and grand-average hydrophathicity (GRAVY).

Peptide	Predicted antigens	MW	IP	SI	SL	AI	GRAVY
E1	HTEQTEEDLRRTLDTLEVQN	2427.57	4.35	91.31 (Unstable)	3.5 h	73.00	−1.545
E2	TAGKPPVWLLIEMDNQPLEL	2264.66	4.14	54.74 (Unstable)	7.2 h	117.00	−0.030
E3	HGQIGFEDNQERAPTEKGST	2201.29	4.83	34.82 **(Stable)**	3.5 h	24.50	−1.600
E4	SGTDRKKLFESFSQGDASVT	2160.33	5.84	12.25 **(Stable)**	1.9 h	39.00	−0.815
E5	QMTLEPNMLTEYRARPLYQP	2193.52	6.07	82.91 (Unstable)	0.8 h	78.00	−0.170

*As of stability index prediction, the predicted epitopes are considered as stable proteins with the set parameters of the tool used.*

### Signal Peptide Analysis

**FIGURE 2:**
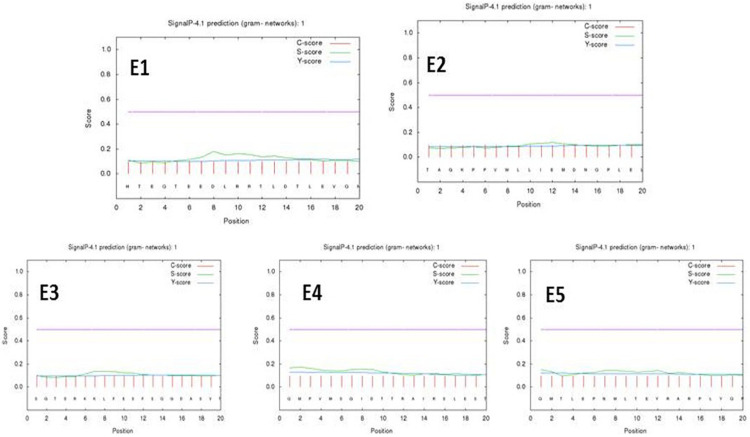
Signal P-noTM neural predictions based on the D-cutoff value using Signal P 4.0 server (C score, S score, and Y score are depicted as pink, green, and blue respectively).

A neural network for transmembrane proteins was provided by the SignalP 4.1 tool for peptides E1–E5. The predicted epitopes did not show any transmembrane signals at a D-cutoff value of 0.51 under a signal-TM neural network. The PSORTb server predicted a final localization score of 7.88 for the gacS protein sequence as a cytoplasmic membrane protein as its localization yielding a preliminary clue toward the drug delivery systems (Figure 2 and Supplementary Table 4).

### Selection of T-Cell Epitopes and Immunogenic Peptides as Analyzed With MHC/HLA Alleles

On the basis of the consensus combinatorial score in the IEDB-AR server, the T-cell epitopes subjected for MHC class I and class II binding predictions yielded immunogenic peptides of varying lengths based on the percentile ranks (≤0.2) eliciting higher affinity under ANN and SMM based IC_50_ values (IC_50_ < 200 nm) (Supplementary Table 5). Binding HLA alleles with T-cell class-I immuno-dominant peptides was also recorded based on their interactions with alleles of significant frequency available as a default in the IEDB server. Further binding affinities between the peptides and T cells, especially associated with T-cell receptors (TCRs), was interpreted based on the class-I immunogenicity prediction scores. With the exception of E4, all the other epitopes yielded a positive score. IEDB conservancy analysis showed 100% conserved sequences within the gacS sequence (Supplementary Table 6).

### MHC Restrictions and Cluster Analysis

**FIGURE 3:**
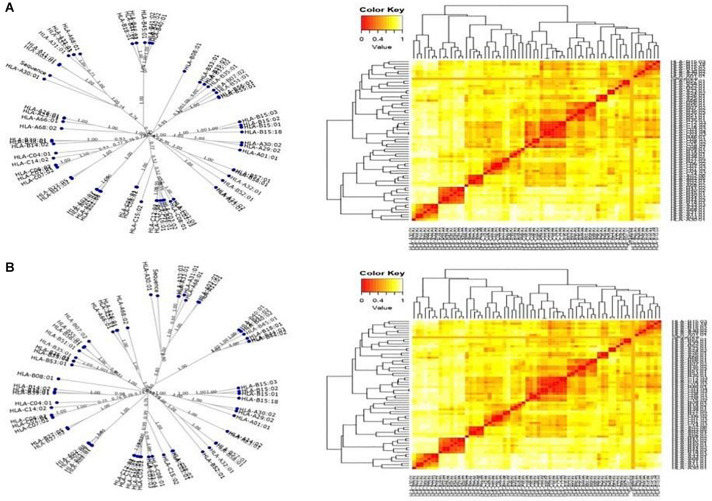
Cluster analysis representing the functional relationships between the predicted peptides with **(A)** MHC class I and **(B)** MHC class II molecules represented by graphical tree and heat map formats with all the available alleles (red zone indicates strong interactions, and yellow zone indicates weak interactions).

The T-cell dominant peptides, as predicted by the IEDB server, analyzed based on the IC_50_ values, were reassessed for their interactions with the HLA alleles by MHC cluster analysis. Dynamic graphical tree and the heat maps were assessed for HLA interactions with the red color indicating strong interactions while yellow showed weaker interactions with appropriate annotations (Figure 3).

### Protein-Peptide Interactions

**FIGURE 4:**
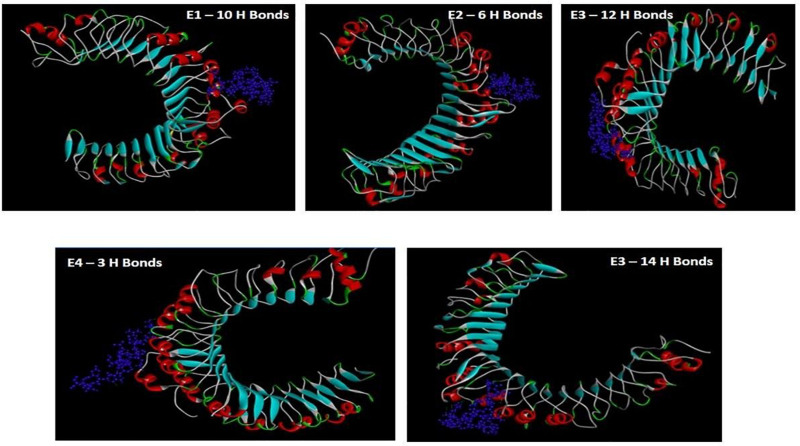
Protein-peptide interaction pictures of the predicted epitopes with TLR-2.

The GalaxyWEB server yielded docked pictures of the immuno-dominant peptides with the TLR-2 receptor suggesting the potency of the chosen epitopes in evoking an immune response. E5 was scored the best interacted peptide, with the highest number of 14 hydrogen bonds. E3 showed 12 hydrogen bonds, followed by E1 with 10 hydrogen bonds. Interactions with E2 and E4 showed only six and three hydrogen bonds respectively (Figure 4).

## Discussion

The present investigation was undertaken as a novel, first-of-its-kind study based on the reverse vaccinology technique in designing novel vaccine candidates targeting the gacS*-*associated virulence factor in the TCS component of *A. baumannii*. Considering prophylaxis as the best alternate strategy for *A. baumannii*, with the advent of bioinformatics, the study was designed as an observational, *in silico* experimentation analysis for the design and evaluation of gacS vaccine candidates for *A. baumannii*. Selection of B-cell and T-cell dominant epitopes is considered a vital step in vaccine development, as they significantly bind with the immunological receptors and are considered to be highly crucial to evoking and eliciting both humoral and cell-mediated immune responses in the host (Yao et al., 2015). Predictions of promiscuous gacS vaccine peptides were thus successfully achieved in the present study by the immuno-informatics approach utilizing the available genomic and proteomic reservoirs under a single computational platform comprising various databases and tools.

A significant approach targeting gacS was made to suggest the considerable relevance in the predictions that can lead to the immunotherapies against the TCS of *A. baumannii*. GgacS TCS with a role in the citrate metabolism of *A. baumannii* and acting as a sensor kinase regulate other vital bacterial component biosynthesis pathways. GacS was thus selected as an antigenic component amid many TCS operons in the present immuno-informatics vaccine peptide construction analysis. Selection of specific proteins from the UniProt database being the initial step in gacS peptide sequence retrieval, it was astonishing to note a single protein with the name gacS was submitted under the name of *BarA* with a unique ID of Q8RMF4 from *A. baumannii* ID 470 with 935aa residues, and FASTA sequences of that protein were thus retrieved for further epitope predictions.

With the goal of predicting promising B-cell epitopes of gacS, online servers such as BCPred server, AAP Prediction tool, BepiPred-2.0 server and ABCPred server were applied. Out of 133 epitopes predicted, in comparison with the tools, 9 epitopes as predicted by the AAP prediction tool were further selected for analysis afterward. This is because the AAP tool predicts B-cell epitopes based on the amino-acid paired (AAP) antigenicity scale in comparison with the sliding window approach of predicted algorithms in other servers and tools, and it is also documented as the superior tool for B-cell epitope predictions (Chen et al., 2007). However, other tools were also employed to assess the epitope overlapping in predictions and also to compare the threshold values scored upon predictions. ABCPred server also predicted a near value of 0.9 in many epitopes with ranking of the epitopes from gacS sequence (Supplementary Table 1), with BCPred records of four epitopes with a threshold value of 0.9 (Supplementary Table 3). BepiPred predictions can be considered in cases where there is a necessity for the minimum and maximum threshold values.

Evaluation of antigenicity was also promising in the present study, as the ANTIGENPro and AlgPred tools yielded five potent antigens E1, E2, E3, E4, and E5. Prediction was based on an independent alignment applying protein data microarrays based on the sequence of five pathogens set as default in the server. The antigenicity of the vaccine peptides was evaluated in VaxiJen server based on the *z* score of various physical and biochemical properties set at a threshold value of 0.4. VaxiJen predicted antigenic peptides based only on the physical and chemical properties of the peptides without the alignment of sequences.

The physicochemical properties of the deduced proteins based on various parameters suggest the promising effects of the predicted antigenic peptides, with two stable peptides E3 and E4. Meanwhile the aliphatic index of the other peptides had shown high thermostability albeit possessing an unstable stability index. The GRAVY values of the peptides with a negative value was deduced based on the standard formula, i.e., the ratio of the sum of the hydropathy values of the amino acids to the number of residues in the sequences. The shelf life recorded by the ProtParam tool was also promising for the epitopes to undergo experimental validations in *in vitro* assessment studies. The shelf life of the peptides was deduced by the server based on the N-terminal rule, in three model organisms, i.e., human, yeast, and *E. coli* (Varshavsky, 1997). E3 and E4 were also considered stable peptides and were evaluated as non-allergenic peptides.

Subcellular localization and prioritization of the target gacS peptides by the PSORTb tool would be yet another crucial step in the optimization of the peptide candidates, minimizing time, labor, and resources. Studies document that the location analysis by suitable tools such as PSORTb, in addition to CELLO, the Swiss-Prot database, and TMHMM tools, may aid in filtering druggable targets based on pathway determinations. In addition, it is also noteworthy to understand that cytoplasmic membrane proteins are suitable for small-molecule drug development while membrane peptides could be utilized for vaccine development (Solanki and Tiwari, 2018). However, the peptides in the present study were not subjected to pathway determinations, except that the protein is localized to cytoplasmic membrane, which is yet another valuable finding in the study.

With the known fact of foreign peptides eliciting both humoral and cell-mediated responses against the pathogens in the host, it is fascinating to note that both T cells and B cells elicit significant response against potent pathogens like *A. baumannii*. In this note, the study had deduced promiscuous T-cell binding epitopes restricted to both MHC class-I and class-II associated HLA alleles. The IEDB server was highly useful in predicting the T-cell dominant immuno-peptide as it involves a consensus apporach in prediction. The output was sorted on the basis of the lower percentile ranks and was associated with the IC_50_ values (<200 nm) under different algorithms such as ANN and SMM scores with ranks. Functional relationships were also successfully assessed in the present investigation as interpreted with the graphical tree and heat maps of the peptides with frequent HLA alleles. In this context, the MHCcluster server was appropriate for algorithmic annotations, indicating the strong and weak interactions of the peptides with the alleles as red and yellow, respectively (Oany, 2017). The protein-peptide complexes observed in the study were interpreted only in terms of the number of hydrogen bonds without further binding energy assessments, also limiting the documentation, with the lacuna of protein modeling, which could not be achieved due to the least number of amino-acid sequences. E3 was considered as the best epitope though E5 yielded a higher number of hydrogen bonds (*n* = 14), as the later (E5) was unstable.

## Conclusion

To conclude, an attempt to predict the few vaccine peptides targeting the gacS*-*mediated TCS in *A. baumannii* was made in the present study by applying reverse vaccinology technology. Diligent applications of the computational tools and databases have increased the probability of finding novel peptide candidates with minimal trials and errors as compared with conventional vaccine preparation protocols. To make further progress from the available gacS vaccine peptide data, it is evidently necessary to design chimeric vaccine constructs together with systemic animal studies.

## Data Availability Statement

All datasets generated for this study are included in the article/Supplementary Material.

## Author Contributions

AS designed the concept and evaluated and drafted the manuscript.

## Conflict of Interest

The author declares that the research was conducted in the absence of any commercial or financial relationships that could be construed as a potential conflict of interest.

## Footnotes

1 https://www.uniprot.org/

2 http://ailab.ist.psu.edu/bcpred/predict.html

## Supplementary Material

TABLE S1 Recurrent neural network prediction of B-cell epitopes from gacS by ABCPred with the rank and score of the predicted peptides.

TABLE S2 Prediction of linear B-cell epitopes using BepiPred software (Threshold value > 0.5).

TABLE S3 B-cell epitope predictions by BCPred software.

TABLE S4 SignalP 4.0 predictions for the transmembrane peptides (D-cutoff signal-noTM networks: 0.57, D-cutoff signal-TM networks: 0.51).

TABLE S5 Consensus method predictions with HLA alleles for T-cell MHC class-I and class-II binding epitopes based on the percentile ranks (Lowest rank) and IC_50_ values (<50 nM, High binding affinity).

TABLE S6 Class-I immunogenicity predictions with positive values indicating a greater probability of evoking an immune response and degree of conservancy of the epitopes within the gacS protein sequence.
