# Supplementary material for: Delineating the Immuno-Dominant Antigenic Vaccine Peptides Against gacS-Sensor Kinase in Acinetobacter baumannii: An in silico Investigational Approach
Source: Front Microbiol. 2020 Sep 8;11:2078. doi: 10.3389/fmicb.2020.02078 (PMC7506167; doi:10.3389/fmicb.2020.02078)
Supplement: TABLE S1 — Recurrent neural network prediction of B-cell epitopes from gacS by ABCPred with the rank and score of the predicted peptides. [file Table_1.DOCX]

**Supplementary table 1: Recurrent neural network prediction of B-cell epitopes from gacS by ABCPred with the rank and score of the predicted peptides**

| Rank | Sequence | Start position | Score |
| --- | --- | --- | --- |
| 1 | QQVIGDALRFKQILTN | 400 | 0.96 |
|  |  |  |  |
| 1 | IGYRDNRYWPNFTQNN | 115 | 0.96 |
|  |  |  |  |
| 2 | DSGIGLSGTDRKKLFE | 451 | 0.94 |
|  |  |  |  |
| 3 | GQTYLSIGYRDNRYWP | 109 | 0.91 |
|  |  |  |  |
| 4 | EQFISTLRKERRRADD | 890 | 0.88 |
|  |  |  |  |
| 4 | FMDIQMPVMSGIDTTR | 719 | 0.88 |
|  |  |  |  |
| 5 | TQSILDLFSRLKHLDQ | 568 | 0.87 |
|  |  |  |  |
| 5 | EEHEIEHPHFEHLQVV | 527 | 0.87 |
|  |  |  |  |
| 6 | LMHGQIGFEDNQERAP | 495 | 0.86 |
|  |  |  |  |
| 6 | VRIIDTAGKPPVWLLI | 146 | 0.86 |
|  |  |  |  |
| 7 | MQLPIIALTAHALADE | 747 | 0.84 |
|  |  |  |  |
| 8 | RLYGATRYVGTPKLQQ | 869 | 0.83 |
|  |  |  |  |
| 8 | EQIIQILTQWTKNNFT | 782 | 0.83 |
|  |  |  |  |
| 8 | MSGIDTTRAIRSLEST | 727 | 0.83 |
|  |  |  |  |
| 8 | TWLIVDHSGDTEALLK | 587 | 0.83 |
|  |  |  |  |
| 8 | MYEIRMQLQRLNADTL | 205 | 0.83 |
|  |  |  |  |
| 9 | HIMQSMFSEKNLKRAA | 88 | 0.81 |
|  |  |  |  |
| 9 | HHVVAEALDPEILNWQ | 805 | 0.81 |
|  |  |  |  |
| 9 | SRRWIAPMYEIRMQLQ | 198 | 0.81 |
|  |  |  |  |
| 10 | TLEPNMLTEYRARPLY | 620 | 0.80 |
|  |  |  |  |
| 10 | AHPATASVLRYYLENY | 546 | 0.80 |
|  |  |  |  |
| 10 | AKSVFLANISHELRTP | 291 | 0.80 |
|  |  |  |  |
| 10 | HIVINSSGELRLLQRD | 223 | 0.80 |
|  |  |  |  |
| 11 | HILAVDDHLPNLIVLE | 666 | 0.79 |
|  |  |  |  |
| 11 | IQLLSDQPIFEEEQQD | 644 | 0.79 |
|  |  |  |  |
| 11 | DGEIIVRVRMEHDDIG | 426 | 0.79 |
|  |  |  |  |
| 12 | RAPTEKGSTFWFTAQF | 508 | 0.78 |
|  |  |  |  |
| 12 | KHIAMAFYYADNIPQQ | 386 | 0.78 |
|  |  |  |  |
| 12 | PIMILTCVGAFLVLTE | 30 | 0.78 |
|  |  |  |  |
| 13 | GTPKLQQVTGDFEQFI | 878 | 0.77 |
|  |  |  |  |
| 13 | QQLIELEDFPQLEHVL | 852 | 0.77 |
|  |  |  |  |
| 13 | EDLAQDLLKMLVDSFP | 830 | 0.77 |
|  |  |  |  |
| 13 | ASAILARYNQIAKDLY | 58 | 0.77 |
|  |  |  |  |
| 13 | TNLISNAIKFTPDGEI | 414 | 0.77 |
|  |  |  |  |
| 13 | AFLVLTETSRSAKQQQ | 39 | 0.77 |
|  |  |  |  |
| 13 | VQNITYRQARDQAISS | 273 | 0.77 |
|  |  |  |  |
| 13 | LKEHTEQTEEDLRRTL | 253 | 0.77 |
|  |  |  |  |
| 14 | TLVELQPDEYDHAQHI | 74 | 0.76 |
|  |  |  |  |
| 14 | LKEIRSRYQGNLAVYG | 601 | 0.76 |
|  |  |  |  |
| 15 | KERRRADDGFIEEVMR | 898 | 0.75 |
|  |  |  |  |
| 15 | KMLVDSFPTELEEMQQ | 838 | 0.75 |
|  |  |  |  |
| 15 | VGMNDYVTKPIQMEQI | 769 | 0.75 |
|  |  |  |  |
| 15 | YNQIAKDLYTLVELQP | 65 | 0.75 |
|  |  |  |  |
| 15 | SVTRQFGGTGLGLAIS | 474 | 0.75 |
|  |  |  |  |
| 16 | GFIEEVMRRFDELGLV | 906 | 0.74 |
|  |  |  |  |
| 16 | QSLQLAANKEDLAQDL | 821 | 0.74 |
|  |  |  |  |
| 16 | HDDIGQCLLHFSVQDS | 437 | 0.74 |
|  |  |  |  |
| 16 | MLSPLAAQKHIAMAFY | 378 | 0.74 |
|  |  |  |  |
| 17 | EALNIIQERIDQKLKP | 699 | 0.73 |
|  |  |  |  |
| 17 | RARPLYQPLSRSGLIQ | 630 | 0.73 |
|  |  |  |  |
| 17 | FFGPISYNHNNIYGVR | 132 | 0.73 |
|  |  |  |  |
| 18 | GNLAVYGYQMTLEPNM | 610 | 0.72 |
|  |  |  |  |
| 19 | KNLKRAALIDSNGQTY | 97 | 0.71 |
|  |  |  |  |
| 19 | AIRSLESTLDGEMQLP | 735 | 0.71 |
|  |  |  |  |
| 19 | KKLFESFSQGDASVTR | 462 | 0.71 |
|  |  |  |  |
| 19 | FSKIDAGKLELETAPF | 351 | 0.71 |
|  |  |  |  |
| 19 | QRLNADTLDQHIVINS | 213 | 0.71 |
|  |  |  |  |
| 20 | LGLVIKEVESAAHQIL | 918 | 0.70 |
|  |  |  |  |
| 20 | RIDQKLKPFDLVFMDI | 707 | 0.70 |
|  |  |  |  |
| 20 | LRQQNLSNEQNLYLQT | 318 | 0.70 |
|  |  |  |  |
| 21 | SGDTEALLKEIRSRYQ | 594 | 0.69 |
|  |  |  |  |
| 22 | IFEEEQQDFNGQGLHI | 652 | 0.68 |
|  |  |  |  |
| 22 | LALINDVLDFSKIDAG | 342 | 0.68 |
|  |  |  |  |
| 23 | FDLEEAVFDVMDMLSP | 366 | 0.67 |
|  |  |  |  |
| 23 | PNFTQNNNFFGPISYN | 124 | 0.67 |
|  |  |  |  |
| 24 | DFPQLEHVLHRLYGAT | 859 | 0.66 |
|  |  |  |  |
| 24 | YYLENYQVPHIETQSI | 556 | 0.66 |
|  |  |  |  |
| 24 | FTAQFAVDEEHEIEHP | 519 | 0.66 |
|  |  |  |  |
| 24 | DGFIHLLLRQQNLSNE | 311 | 0.66 |
|  |  |  |  |
| 25 | NVKTTKALSGQEALNI | 688 | 0.65 |
|  |  |  |  |
| 25 | LELETAPFDLEEAVFD | 359 | 0.65 |
|  |  |  |  |
| 25 | GQLIALIFVPIMILTC | 21 | 0.65 |
|  |  |  |  |
| 25 | KPPVWLLIEMDNQPLE | 154 | 0.65 |
|  |  |  |  |
| 26 | TLSKRLRLNHAYGQLI | 9 | 0.64 |
|  |  |  |  |
| 26 | HPHFEHLQVVSYLAHP | 533 | 0.64 |
|  |  |  |  |
| 26 | FSQGDASVTRQFGGTG | 468 | 0.64 |
|  |  |  |  |
| 27 | NLIVLEALLGELNVKT | 676 | 0.63 |
|  |  |  |  |
| 28 | SGELRLLQRDIANVVK | 229 | 0.62 |
|  |  |  |  |
| 28 | NQPLELARYRILIALV | 165 | 0.62 |
|  |  |  |  |
| 29 | VFDVMDMLSPLAAQKH | 372 | 0.61 |
|  |  |  |  |
| 30 | RQARDQAISSNQAKSV | 279 | 0.60 |
|  |  |  |  |
| 31 | SRSAKQQQLHHASAIL | 47 | 0.59 |
|  |  |  |  |
| 32 | LHFSFLELKEHTEQTE | 246 | 0.57 |
|  |  |  |  |
| 33 | HELRTPLNSIDGFIHL | 301 | 0.56 |
|  |  |  |  |
| 33 | EEDLRRTLDTLEVQNI | 261 | 0.56 |
|  |  |  |  |
| 34 | DEKQKLLKVGMNDYVT | 761 | 0.55 |
|  |  |  |  |
| 34 | QPLSRSGLIQLLSDQP | 636 | 0.55 |
|  |  |  |  |
| 35 | GLAISKQLVHLMHGQI | 485 | 0.54 |
|  |  |  |  |
| 36 | QTIRKSSAHLLALIND | 332 | 0.53 |
