# Supplementary material for: Delineating the Immuno-Dominant Antigenic Vaccine Peptides Against gacS-Sensor Kinase in Acinetobacter baumannii: An in silico Investigational Approach
Source: Front Microbiol. 2020 Sep 8;11:2078. doi: 10.3389/fmicb.2020.02078 (PMC7506167; doi:10.3389/fmicb.2020.02078)
Supplement: TABLE S2 — Prediction of linear B-cell epitopes using BepiPred software (Threshold value > 0.5). [file Table_2.DOCX]

**Supplementary table 2: Prediction of linear B-cell epitopes using BepiPred software**

**(Threshold value >0.5)**

| **Peptide Start sequence** | **Peptide End sequence** | **Epitope predicted** |
| --- | --- | --- |
| 5 | 16 | NKTLSKRLRLNH |
| 47 | 57 | SAKQQQLHHAS |
| 65 | 69 | QIAKD |
| 75 | 85 | ELQPDEYDHAQ |
| 114 | 135 | GYRDNRYWPNFTQNNNFFGPIS |
| 213 | 229 | LNADTLDQHIVINSSGE |
| 245 | 282 | HFSFLELKEHTEQTEEDLRRTLDTLEVQNITYRQARDQ |
| 467 | 479 | SQGDASVTRQFGG |
| 503 | 511 | NQERAPTEK |
| 523 | 534 | EEHEIEHPHF |
| 647 | 663 | DQPIFEEEQQDFNGQGL |
| 706 | 713 | QERIDQKLK |
| 742 | 746 | STLDG |
| 758 | 787 | LADEKQKLLKVGMNDYVTKPIQMEQIIQ |
| 790 | 832 | WTKNNFTAQNLAKDHHVVAEALDPEILNWQQSLQLAANKED |
| 846 | 861 | TELEEMQQLIELEDFP |
| 895 | 917 | TLRKERRRADDGFIEEVMRRFD |
| 928 | 933 | AAHQIL |
