# Supplementary material for: Delineating the Immuno-Dominant Antigenic Vaccine Peptides Against gacS-Sensor Kinase in Acinetobacter baumannii: An in silico Investigational Approach
Source: Front Microbiol. 2020 Sep 8;11:2078. doi: 10.3389/fmicb.2020.02078 (PMC7506167; doi:10.3389/fmicb.2020.02078)
Supplement: TABLE S3 — B-cell epitope predictions by BCPred software. [file Table_3.DOCX]

**Supplementary table 3: B-cell epitope predictions by BCPred software**

| Position | Epitope | Score |
| --- | --- | --- |
| 893 | TLRKERRRADDGFIEEVMRR | 0.995 |
| 250 | ELKEHTEQTEEDLRRTLDTL | 0.972 |
| 495 | HGQIGFEDNQERAPTEKGST | 0.943 |
| 518 | TAQFAVDEEHEIEHPHFEHL | 0.928 |
| 75 | ELQPDEYDHAQHIMQSMFSE | 0.898 |
| 114 | GYRDNRYWPNFTQNNNFFGP | 0.845 |
| 271 | VQNITYRQARDQAISSNQAK | 0.844 |
| 544 | AHPATASVLRYYLENYQVPH | 0.833 |
| 465 | SFSQGDASVTRQFGGTGLGL | 0.827 |
| 318 | QQNLSNEQNLYLQTIRKSSA | 0.822 |
| 611 | AVYGYQMTLEPNMLTEYRAR | 0.82 |
| 581 | QKDNTWLIVDHSGDTEALLK | 0.805 |
| 840 | DSFPTELEEMQQLIELEDFP | 0.794 |
| 776 | PIQMEQIIQILTQWTKNNFT | 0.774 |
| 720 | IQMPVMSGIDTTRAIRSLES | 0.763 |
| 420 | KFTPDGEIIVRVRMEHDDIG | 0.755 |
