# Supplementary material for: Delineating the Immuno-Dominant Antigenic Vaccine Peptides Against gacS-Sensor Kinase in Acinetobacter baumannii: An in silico Investigational Approach
Source: Front Microbiol. 2020 Sep 8;11:2078. doi: 10.3389/fmicb.2020.02078 (PMC7506167; doi:10.3389/fmicb.2020.02078)
Supplement: TABLE S4 — SignalP 4.0 predictions for the transmembrane peptides (D-cutoff signal-noTM networks: 0.57, D-cutoff signal-TM networks: 0.51). [file Table_4.DOCX]

**Supplementary table 4: SignalP 4.0 predictions for the transmembrane peptides [D-cut off signal-noTM networks: 0.57, D-cut off signal-TM networks: 0.51]**

| **Peptide** | **Predicted antigens** | **Score** | **Signal TM** |
| --- | --- | --- | --- |
|  | HTEQTEEDLRRTLDTLEVQN | 0.122 | No |
|  | TAGKPPVWLLIEMDNQPLEL | 0.095 | No |
|  | HGQIGFEDNQERAPTEKGST | 0.113 | No |
|  | SGTDRKKLFESFSQGDASVT | 0.108 | No |
|  | QMTLEPNMLTEYRARPLYQP | 0.122 | No |
