# Supplementary material for: Delineating the Immuno-Dominant Antigenic Vaccine Peptides Against gacS-Sensor Kinase in Acinetobacter baumannii: An in silico Investigational Approach
Source: Front Microbiol. 2020 Sep 8;11:2078. doi: 10.3389/fmicb.2020.02078 (PMC7506167; doi:10.3389/fmicb.2020.02078)
Supplement: TABLE S5 — Consensus method predictions with HLA alleles for T-cell MHC class-I and class-II binding epitopes based on the percentile ranks (Lowest rank) and IC50 values (<50 nM, High binding affinity). [file Table_5.DOCX]

**Supplementary table 5: Consensus method predictions with HLA alleles for T-cell MHC class-I and class-II binding epitopes based on the percentile ranks (Lowest rank) and IC_50_ values (<50nM – High binding affinity)**

| **T cell MHC class I HLA alleles and binding peptides** | | | | | | | | | | | | | | |
| --- | --- | --- | --- | --- | --- | --- | --- | --- | --- | --- | --- | --- | --- | --- |
| *Alleles* | *Peptide*  *sequences* | *Start* | *End* | *Length* | *Peptide* | *Percentile rank* | *ANN*  *IC_50_* | *ANN rank* | *SMM*  *IC_50_* | *SMM*  *rank* | *Comb*  *Lib score* | *Comb*  *Lib rank* | *NetMHC*  *pan score* | *NetMHC pan rank* |
| HLA-*40:01 | HTEQTEEDLRRTLDTLEVQN | 5 | 13 | 9 | TEEDLRRT | 0.41 | 42.55 | 0.13 | 86.28 | 0.7 | - | - | _-_ | _-_ |
| HLA-*40:01 | TAGKPPVWLLIEMDNQPLEL | 11 | 20 | 10 | IEMDNQPLEL | 0.21 | 100.27 | 0.23 | 40.18 | 0.2 | - | - | _-_ | _-_ |
| HLA-*30:01 | HGQIGFEDNQERAPTEKGST | 6 | 13 | 9 | FEDNQERA | 0.1 | 2.26 | 0.02 | 3.58 | 0.1 | 1.55E-05 | 0.4 | - | - |
| HLA-*68:02 | SGTDRKKLFESFSQGDASVT | 10 | 19 | 10 | ESFSQGDASV | 0.48 | 30.24 | 0.27 | 34.22 | 0.7 | - | - | - | - |
| HLA-*01:01 | QMTLEPNMLTEYRARPLYQP | 3 | 12 | 10 | TLEPNMLTEY | 0.18 | 64.72 | 0.17 | 237.03 | 0.2 | - | - | - | - |
| **T cell MHC class II HLA alleles and binding peptides** | | | | | | | | | | | | | | |
| HLA-DRB1*03:01 | HTEQTEEDLRRTLDTLEVQN | 6 | 20 | 15 | EEDLRRTLDTLEVQN | 28 | 1228.5 | 28 | 1753 | 26 | - | - | - | - |
| HLA-DRB4*01:01 | TAGKPPVWLLIEMDNQPLEL | 4 | 18 | 15 | KPPVWLLIEMDNQPL | 2.5 | 44.2 | 2.1 | 129 | 2.5 | 4915.23 | 57 | - | - |
| HLA-DRB1*03:01 | HGQIGFEDNQERAPTEKGST | 2 | 16 | 15 | GQIGFEDNQERAPTE | 33 | 1595.5 | 32 | 2308 | 33 | - | - | - | - |
| HLA-DRB1*15:01 | SGTDRKKLFESFSQGDASVT | 4 | 18 | 15 | DRKKLFESFSQGDAS | 30 | 918 | 30 | 553.6 | 26 | - | - | - | - |
| HLA-DRB1*15:01 | QMTLEPNMLTEYRARPLYQP | 5 | 19 | 15 | EPNMLTEYRARPLYQ | 3.5 | 44.5 | 3 | 126 | 3.5 | - | - | - | - |
