# Supplementary material for: Delineating the Immuno-Dominant Antigenic Vaccine Peptides Against gacS-Sensor Kinase in Acinetobacter baumannii: An in silico Investigational Approach
Source: Front Microbiol. 2020 Sep 8;11:2078. doi: 10.3389/fmicb.2020.02078 (PMC7506167; doi:10.3389/fmicb.2020.02078)
Supplement: TABLE S6 — Class-I immunogenicity predictions with positive values indicating a greater probability of evoking an immune response and degree of conservancy of the epitopes within the gacS protein sequence. [file Table_6.DOCX]

**Supplementary table 6: Class I immunogenicity predictions with positive values indicating a greater probability of evoking an immune response and degree of conservancy of the epitopes within the gacA protein sequence**

| **Peptide** | **Predicted peptides**  **[E1-E5]** | **Peptide length** | **Score** | **Degree of Conservancy** |
| --- | --- | --- | --- | --- |
| E1 | HTEQTEEDLRRTLDTLEVQN | 20 | 0.39735 | 100% |
| E2 | TAGKPPVWLLIEMDNQPLEL | 20 | 0.004 | 100% |
| E3 | HGQIGFEDNQERAPTEKGST | 20 | 0.28276 | 100% |
| E4 | SGTDRKKLFESFSQGDASVT | 20 | -0.47905 | 100% |
| E5 | QMTLEPNMLTEYRARPLYQP | 20 | 0.0786 | 100% |
